# Supplementary figures and images for: Druggable redox pathways against Mycobacterium abscessus in cystic fibrosis patient-derived airway organoids
Source: PLoS Pathog. 2023 Aug 24;19(8):e1011559. doi: 10.1371/journal.ppat.1011559 (PMC10449475; doi:10.1371/journal.ppat.1011559)

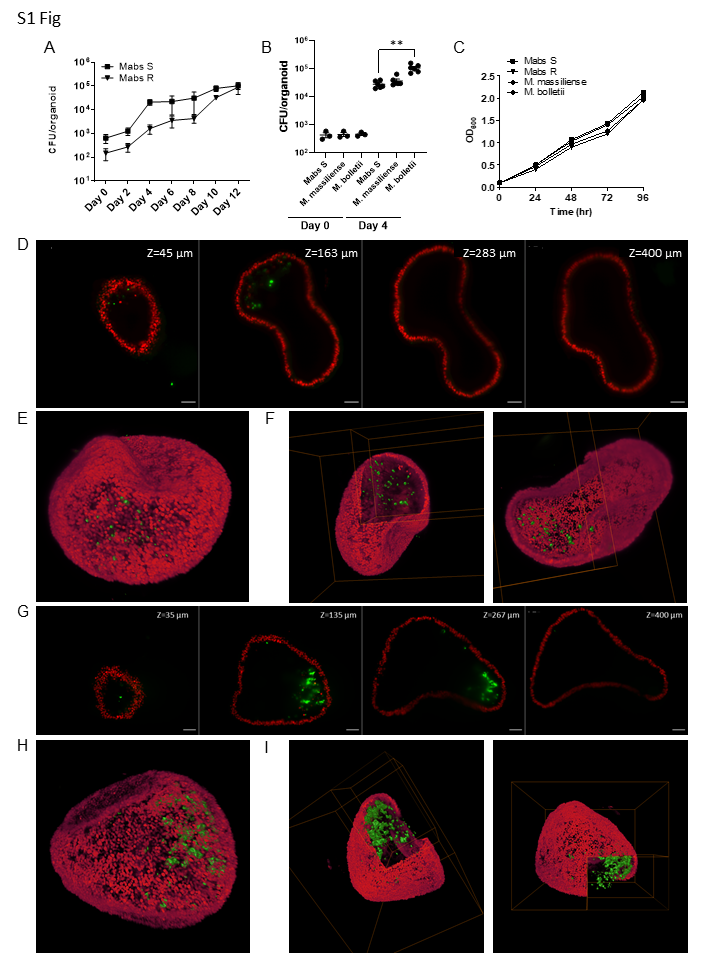

Supplement: S1 Fig — (A) Kinetics of Mabs S and R growth in H-AO. Graph shows three pooled independent experiments. (B) Growth of Mycobacterium abscessus subspecie abscessus (Day 0 n = 3; Day 4 n = 6), subspecie massiliense (Day 0 n = 3; Day 4 n = 6), and subspecie bolletii (Day 0 n = 3; Day 4 n = 6) in H-AO. Graph shows means ± SEM from two independent experiments. Each dot represents one organoid. **P<0.01 by Mann-Whitney test. (C) Kinetics of in vitro growth of Mycobacterium abscessus subspecie abscessus, subspecie massiliense, and subspecie bolletii. Graph represents means from one experiment performed in triplicates. (D-I) 3D light-sheet imaging of airway organoids infected with wasabi (green) Mabs S or R. H-AOs were fixed then stained with propidium iodide to visualize cell nuclei (red) before imaging using Zeiss Lightsheet 1 microscope. (D) XY planes at the indicated z positions of the 400 μm z-stack of an H-AO after infection with Mabs S shown in S1 Movie (10X objective). (E) 3D visualization using AMIRA software of the z-stack of AO after infection with Mabs S. (F) Corner cut from two different angles using AMIRA software through a volume rendering of the nuclei while keeping the Mabs S fluorescent signal. Scale bar: 50 μm. (G) XY planes at the indicated z positions of the 400 μm z-stack of a H-AO after infection with Mabs R shown in S2 Movie (10X objective). (H) 3D visualization using AMIRA software of the z-stack of AO after infection with Mabs R. (I) Corner cut from two different angles using AMIRA software through a volume rendering of the nuclei while keeping the Mabs R fluorescent signal. Scale bar: 50 μm. (TIF) [file ppat.1011559.s002.tif]

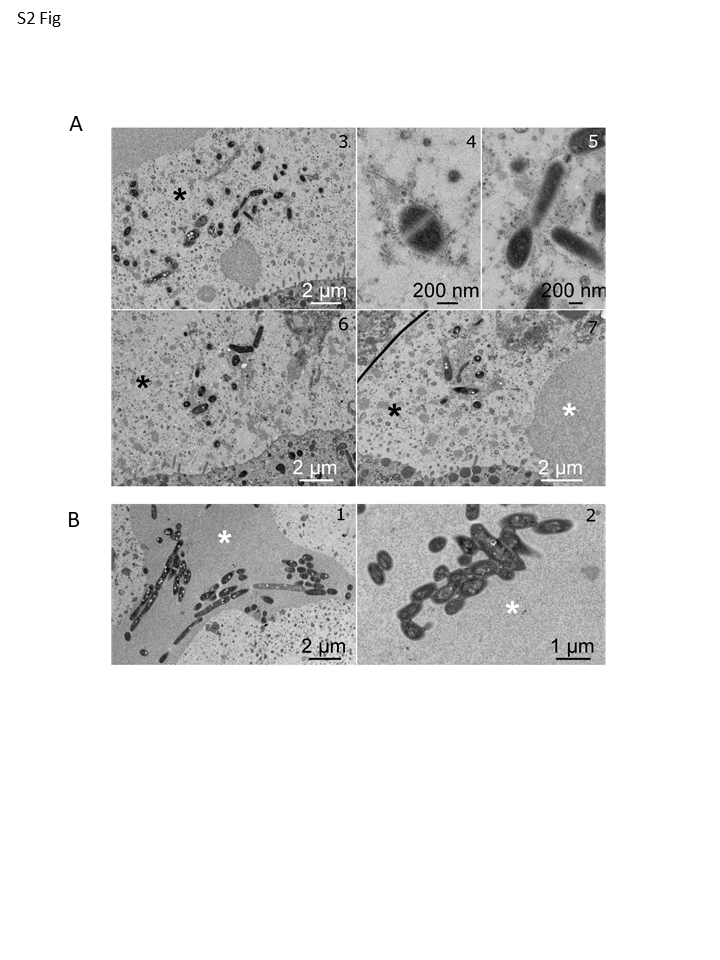

Supplement: S2 Fig — (A) Rows 1 and 2: Transmission electron micrographs of healthy AO infected with Mab-S. Bacteria were found dispersed in loose aggregates in the layer close to the luminal side of the lung epithelium (black asterisk) and excluded from the mucus (white asterisk). Bacteria were not in direct contact with each other and did not show any preferred orientation. An accumulation of fibril-granular material was observed around the aggregates. (B) Transmission electron micrographs of healthy AO infected with Mab-R. Bacteria were found exclusively in the mucus (white asterisk) organized in bundles showing individual cells oriented and in close apposition with each other inside the same bundle. (TIF) [file ppat.1011559.s003.tif]

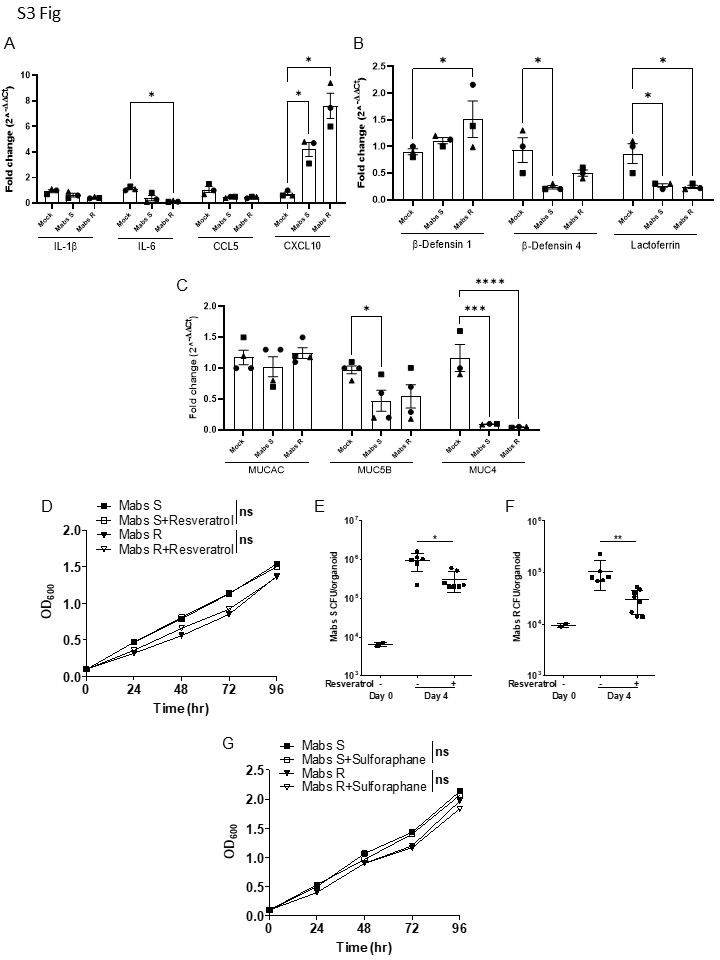

Supplement: S3 Fig — (A-C) Expression pattern of inflammatory cytokines (A), antimicrobial peptides (B), and mucins (C) in mock-infected H-AO, or H-AO infected with Mabs S or R for 4 days. Graphs represent means ± SEM from at least three independent experiments, performed in triplicates. *P<0.05; ***P<0.001; ****P<0.0001 by unpaired T test. (D) Kinetics of in vitro Mabs S and R growth in absence or presence of 10μM resveratrol. Graph represents means from one experiment performed in triplicates. ns = not significative by Mann-Whitney test. (E-F) Bacterial load by CFU assay of H-AO pre-treated with (+) or without (-) 10μM resveratrol for 1hr before infection with Mabs S (E) (n+ = 7; n- = 6) or R (F) (n+ = 8; n- = 6) for 4 days. Graphs represent means ± SD from at two independent experiments, indicated by different symbols. Each dot represents one organoid. *P<0.05; **P<0.01 by Mann-Whitney test. (G) Kinetics of in vitro Mabs S and R growth in absence or presence of 10μM sulforaphane. Graph represents means from one experiment performed in triplicates. ns = not significative by Mann-Whitney test. (TIF) [file ppat.1011559.s004.tif]

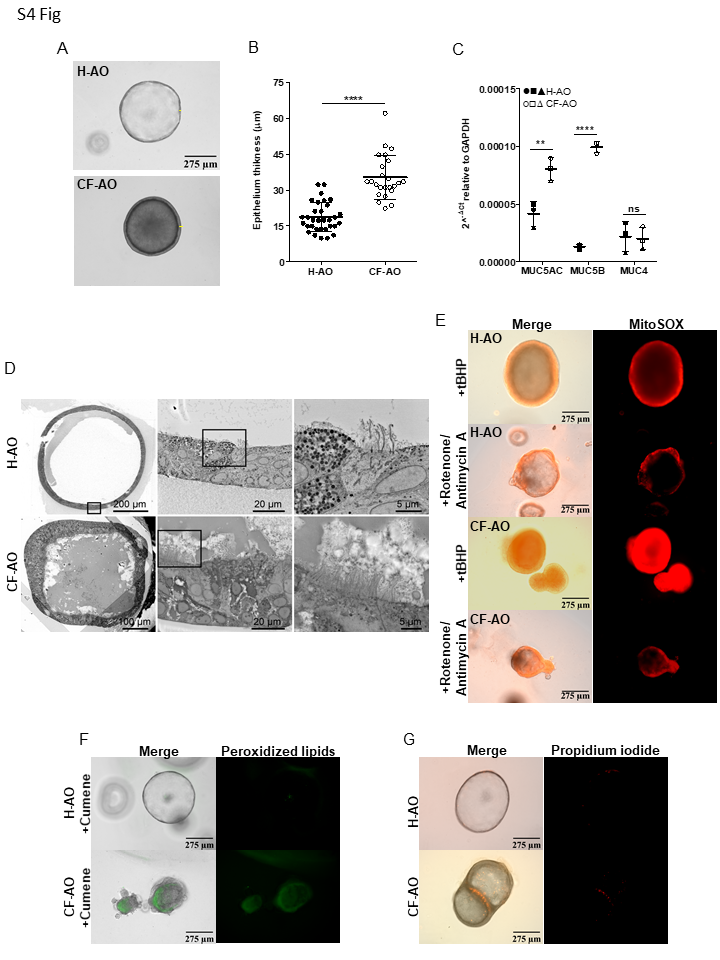

Supplement: S4 Fig — (A, B) Representative bright-field images (A) and quantification (B) of epithelium thickness in healthy AOs (H-AO n = 32) and cystic fibrosis AOs (CF-AO n = 24). Data from three independent wells per donor. (C) Basal expression of mucin genes in H-AO and CF-AO. Graph represents means from three pooled independent experiments, performed in triplicates. **P<0.01; **** P<0.0001; ns = not significative by unpaired T test. (D) Electron micrographs of H-AO and CF-AO revealing mucus accumulation in the lumen and longer cilia in the CF ones. (E) Representative images of mitochondrial ROS production (5μM MitoSOX) in H-AO and CF-AO after 1hr treatment with 20Mm tBHP or a mix of 5μM rotenone and 5μM antimycin A. (F) Representative images of peroxidized lipids (2μM BODIPY) in H-AO and CF-AO after 2hr treatment with 800μM cumene hydroperoxide. (G) Representative images of the basal propidium iodide incorporation (50 μg ml-1) in H-AO and CF-AO. (TIF) [file ppat.1011559.s005.tif]

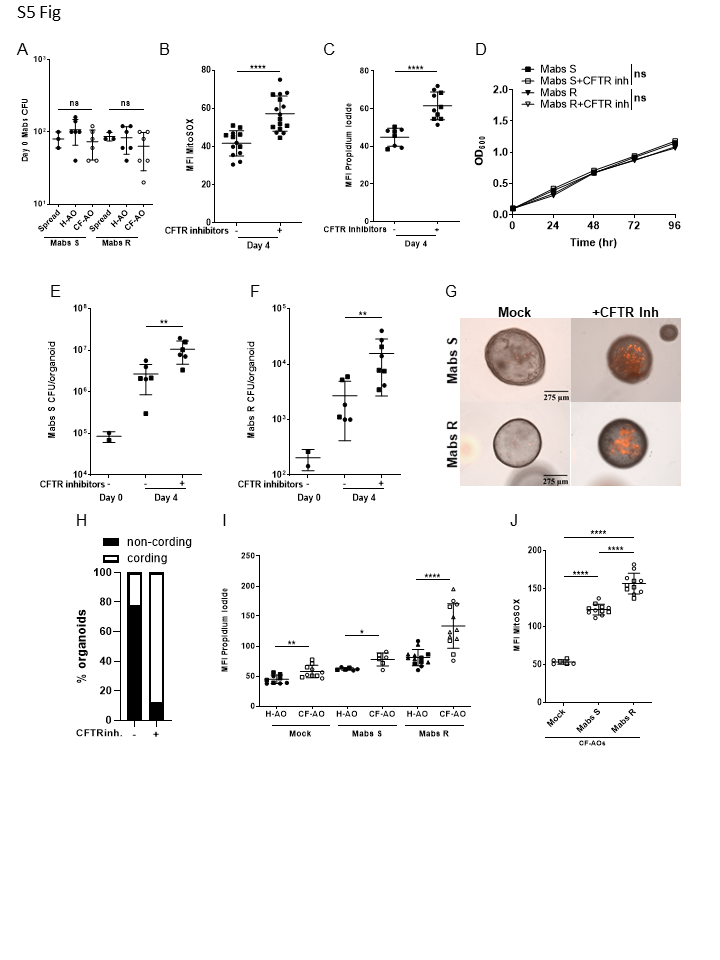

Supplement: S5 Fig — (A) Initial bacterial load evaluation by CFU assay of H-AO and CF-AO infected with Mabs S (H-AO n = 6; CF-AO n = 6) or Mabs R (H-AO n = 6; CF-AO n = 6). (B) MFI quantification of mitochondrial ROS production (5μM MitoSOX) in H-AO after 4 days of being treated with (+ n = 16) or without (- n = 13) 25μM CFTR inhibitors (CFTRinh-172 and GlyH 101). (C) MFI quantification of propidium iodide incorporation (50 μg ml-1) in H-AO after 4 days of being treated with (+ n = 10) or without (- n = 8) 25μM CFTR inhibitors. (D) Kinetics of Mabs S and R growth in absence or presence of 25μM CFTR inhibitors. Data from one experiment performed in triplicates. (E, F) Bacterial load by CFU assay of H-AO pre-treated with (+) or without (-) 25μM CFTR inhibitors for 2 days before infection with Mabs S (E) (n+ = 6; n- = 6) or R (F) (n+ = 8; n- = 6) for 4 days. (G) Representative images of H-AO pre-treated or not with 25μM CFTR inhibitors for 2 days before infection with Mabs S or R for 4 days. (H) Mean percentage of H-AO untreated (n = 18) or treated (n = 30) with 25μM CFTR inhibitors exhibiting cords after 4 days of infection with Mabs R. (I) MFI quantification of propidium iodide incorporation (50 μg ml-1) in Mock-infected H-AO and CF-AO (H-AO n = 9; CF-AO n = 10) or H-AO and CF-AO infected with Wasabi-labelled Mabs S (H-AO n = 6; CF-AO n = 6) or Mabs R (H-AO n = 13; CF-AO n = 12) for 4 days. (J) MFI quantification of mitochondrial ROS production (5μM MitoSOX) in Mock-infected CF-AO (CF-AO n = 6) or CF-AO infected with Wasabi-labelled Mabs S (CF-AO n = 11) or Mabs R (CF-AO n = 11) for 3 days. Except otherwise stated, graphs represent means ± SD from at least two independent experiments indicated by different symbols. Each dot represents one organoid. *P<0.05; **P<0.01; ****P<0.0001; ns = not significative by Mann-Whitney test. (TIF) [file ppat.1011559.s006.tif]

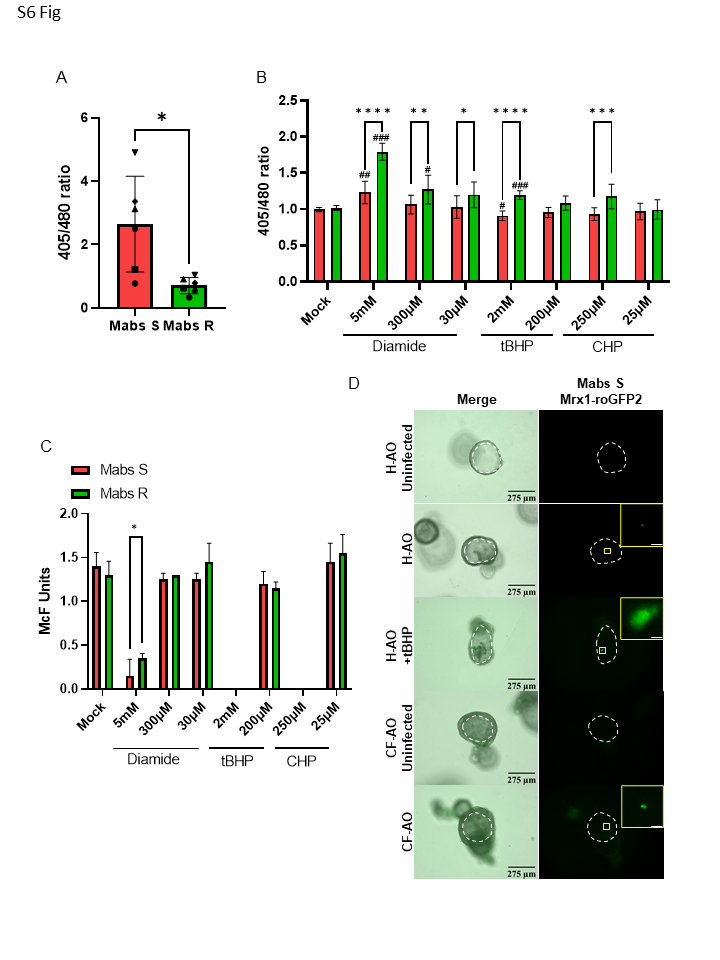

Supplement: S6 Fig — (A) Basal ratiometric sensor response of Mrx1-roGFP2-expressing Mabs S and R measured. * P< 0.05, paired t test of three independent experiments. (B) Mabs S (red) and R (green) expressing Mrx1-roGFP2 were either left untreated (Mock) or exposed to different concentrations of diamide, tert-Butyl hydroperoxide (tBHP) or cumene hydroperoxide (CHP), and the ratiometric sensor response (405/480 ratio) was measured after 2 h post-exposure. Data represent the Mean± SD of three independent experiments. For S to R comparison, Mock condition was normalized to 1. S to R comparison: * P<0.05, ** P<0.01, *** P<0.001, **** P< 0.0001; Mabs variant to their respective Mock control: # P<0.05, ## P< 0.01, ### P< 0.001. (C) Mabs S (red) and R (green) expressing Mrx1-roGFP2 were either left untreated (Mock) or exposed to different concentrations of diamide, tert-Butyl hydroperoxide (tBHP) or cumene hydroperoxide (CHP), and bacterial growth in untreated (Mock) or oxidative agent-treated conditions was measured by the McFarland technique measuring bacterial culture turbidity. (D) Representative images of H-AO and CF-AO treated or not with 200μM tBHP for 1 hour before infection with roGFP2-expressing Mabs S for 4 days. (TIF) [file ppat.1011559.s007.tif]

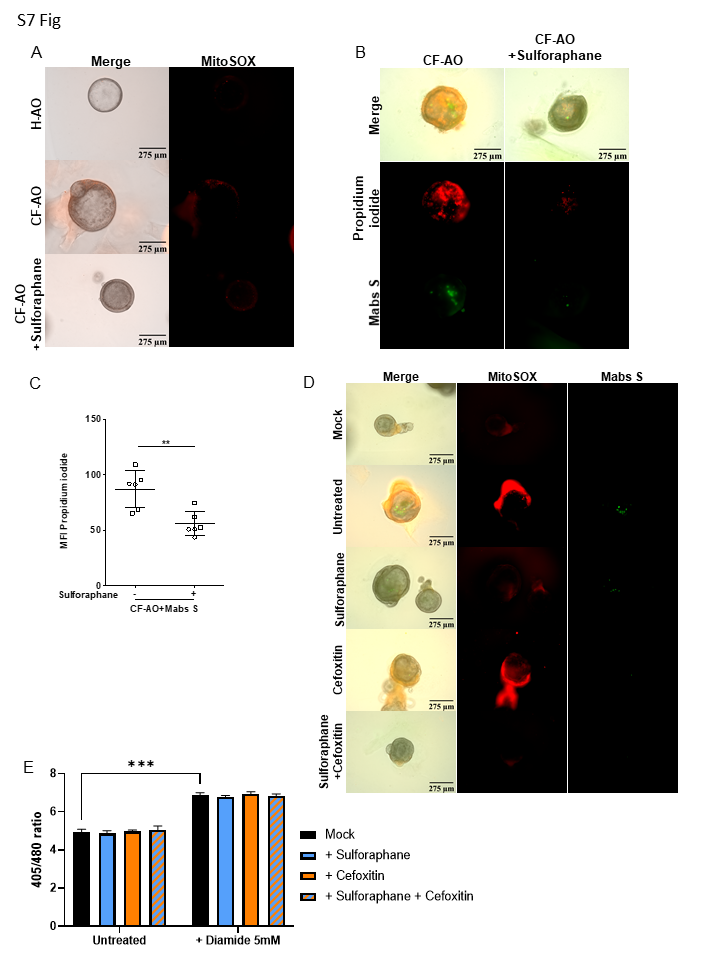

Supplement: S7 Fig — (A) Representative images of mitochondrial ROS production (5μM MitoSOX) in H-AO and CF-AO after 4 days of being treated or not with 10μM sulforaphane. (B, C) Representative images (B) and MFI quantification (C) of propidium iodide incorporation (50 μg ml-1) in CF-AO pre-treated with (+) or without (-) 10μM sulforaphane for 6 hr before infection with Wasabi-labelled Mabs S (n+ = 6; n- = 6) for 4 days. (D) Representative images of MitoSOX staining in Mock- or Mabs-infected CF organoids treated or not with sulforaphane and cefoxitin, alone or in combination. (E) Mabs S expressing Mrx1-roGFP2 was either left untreated (Mock) or exposed to 5mM diamide, then left untreated or treated with 10μM sulforaphane and/or 20μg/ml cefoxitin, and the ratiometric sensor response (405/480 ratio) was measured after 2 h post-exposure. Data represent the Mean± SD of three independent experiments. * P<0.05, by Two-way ANOVA. Except otherwise stated, graphs represent means ± SD from at least two independent experiments, indicate them by different symbols. Each dot represents one organoid. **P<0.01; ***P<0.001 by Mann-Whitney test. (TIF) [file ppat.1011559.s008.tif]
