# Supplementary material for: Druggable redox pathways against Mycobacterium abscessus in cystic fibrosis patient-derived airway organoids
Source: PLoS Pathog. 2023 Aug 24;19(8):e1011559. doi: 10.1371/journal.ppat.1011559 (PMC10449475; doi:10.1371/journal.ppat.1011559)
Supplement: S1 Table — (DOCX) [file ppat.1011559.s011.docx]

**S1 Table. Characteristics of the CF patients**

| **Donor** | **Sex** | **Age (years)** | **Mutation** |
| --- | --- | --- | --- |
| **CF01** | Female | 30 | Class I  G542X/1811+1.6kbA-->G |
| **CF02** | Male | 33 | Class II  ∆F508/∆F508 |
| **CF03** | Male | 28 | Class II  ∆F508/4005+1 G>A |
