# Supplementary material for: Druggable redox pathways against Mycobacterium abscessus in cystic fibrosis patient-derived airway organoids
Source: PLoS Pathog. 2023 Aug 24;19(8):e1011559. doi: 10.1371/journal.ppat.1011559 (PMC10449475; doi:10.1371/journal.ppat.1011559)
Supplement: S2 Table — (DOCX) [file ppat.1011559.s012.docx]

**S2 Table. List of primers used for RT-qPCR**

| **Gene** | **Primers 5’-3’** | **Reference** |
| --- | --- | --- |
| **GAPDH**  **(NM_002046)** | F: CTCCAAAATCAAGTGGGGCGATG  R: GGCATTGCTGATGATCTTGAGGC | [4] |
| **NOX1**  **(NM_007052.5)** | F: TTGTTTGGTTAGGGCTGAATGT  R: GCCAATGTTGACCCAAGGATTTT | PrimerBank |
| **DUOX1**  **(NM_017434.5)** | F: TTCACGCAGCTCTGTGTCAA  R: AGGGACAGATCATATCCTGGCT | [8] |
| **NRF2**  **(NM_006164.5)** | F: TCAGCGACGGAAAGAGTATGA  R: CCACTGGTTTCTGACTGGATGT | PrimerBank |
| **NQO1**  **(NM_000903.3)** | F: CAGACGCCCGAATTCAAATC  R: AGGCTGCTTGGAGCAAAATACA | [9] |
| **HMOX1**  **(NM_002133.3)** | F: TCCGATGGGTCCTTACACTC  R: TAAGGAAGCCAGCCAAGAGA | [9] |
| **IL-1β**  **(NM_000576)** | F: AGCTACGAATCTCCGACCAC  R: GGGAAAGAAGGTGCTCAGGTC | [4] |
| **IL-6**  **(NM_000600.5)** | F: ACTCACCTCTTCAGAACGAATTG  R: CCATCTTTGGAAGGTTCAGGTTG | PrimerBank |
| **CCL5**  **(NM_002985)** | F: CCTCATTGCTACTGCCCTCT  R: CGGGTGACAAAGACGACTGC | [4] |
| **CXCL10**  **(NM_001565)** | F: GTGGCATTCAAGGAGTACCTC  R: GATTCAGACATCTCTTCTCACCC | [4] |
| **β-Defensin 1**  **(NM_005218)** | F: ATGGCCTCAGGTGGTAACTTTC  R: GGTCACTCCCAGCTCACTTG | [4] |
| **β-Defensin 4**  **(NM_080389.3)** | F: TGCCGGAAGAAATGTCGCA  R: CGACTCTAGGGACCAGCAC | [4] |
| **Lactoferrin**  **(NM_002343)** | F: CCCCTACAAACTGCGACCTG  R: CAGACCTTGCAGTTCGTTCAG | [4] |
| **MUC5AC (NM_001304359.2)** | F: GGAACTGTGGGGACAGCTCTT  R: GTCACATTCCTCAGCGAGGTC | [10] |
| **MUC5B**  **(NM_002458.3)** | F: GCCCACATCTCCACCTATGAT  R: GCAGTTCTCGTTGTCCGTCA | PrimerBank |
| **MUC4**  **(NM_018406.7)** | F: CTCAGTACCGCTCCAGCAG  R: CCGCCGTCTTCATGGTCAG | [4] |
