# Supplementary material for: Druggable redox pathways against Mycobacterium abscessus in cystic fibrosis patient-derived airway organoids
Source: PLoS Pathog. 2023 Aug 24;19(8):e1011559. doi: 10.1371/journal.ppat.1011559 (PMC10449475; doi:10.1371/journal.ppat.1011559)
Supplement: Attachment — Submitted filename: PPATHOGENS-D-22-02078-RebuttalLetter.docx [file ppat.1011559.s013.docx]

Manuscript PPATHOGENS-D-22-02078

“Druggable redox pathways against *Mycobacterium abscessus* in cystic fibrosis patient-derived airway organoids’’

Leon Icaza *et al.*

The authors need to ensure that differences in bacterial burden at the time of measurement of experimental parameters is not skewing data interpretation. The authors focused on the oxidative environment without necessarily providing data to measure the oxidative response and do explore the mechanistic basis for the differential virulence. It is important to address the concern regarding microinjection efficiency of healthy versus cystic fibrosis organoids.

Reviewer's Responses to Questions

**Part I – Summary**

Please use this section to discuss strengths/weaknesses of study, novelty/significance, general execution and scholarship.

Reviewer #1: Leon-Icaza et al. develop and exploit human airways organoids as a model system to characterize host-pathogen interaction with an emphasis on the emerging pathogen Mycobacterium abscessus. On the one hand the authors investigate two well described morphotypes of M. abscessus, namely a smooth morphotype and the rough morphotype. The smooth morphotype is covered by a layer of glycopeptido lipids, while these surface molecules are missing/mislocated in the rough morphotype. Upon infection of airway organoids bacteria of both morphotypes multiply (over 12 days) and can trigger an oxidative stress response in the host which helps to control intracellular bacteria but also negatively affects viability of host cells. In the organoids, both morphotypes grow extra-cellularly but form either biofilm like-structure (smooth) or cords (rough), respectively. The R morphotype induces higher levels of oxidative stress. Organoids were derived from people suffering from Cystic Fibrosis and people with unrelated disease. Upon infection of CF-derived organoids a higher oxidative stress response is induced. A drug-mediated suppression of the oxidative stress response inhibits multiplication of the bacteria and favors the antibacterial effects of the b-lactam antibiotic cefoxitin, a first-line drug for the treatment of pulmonary M. abscessus infections.
The investigations demonstrate that the airway organoids model can mimic relevant in vivo features of a human disease (Cytic Fibrosis) and host-pathogen interaction. It provides the possibility to explore new antibacterial and host-directed therapies for an infection posed by a serious and hard-to-treat pathogen. The model may be run at a “medium through-put” size and significantly contribute to the 3R criteria in order to reduce animal experimentation.

In summary, the investigations demonstrate the model can recapitulate important features of host-pathogen interaction and may provide new insights into virulence mechanisms and hint to new therapeutic approaches.

Criticism: The entire investigation is conducted with one (set) of strains, namely M. abscessus type strain. It would be recommendable to extend the investigation to members of the other subspecies and to strains which show increased/decreased susceptibility to the drug(s) used for treatment.

*The authors thank the reviewer for his/her fair and constructive evaluation of our study. In order to address the points raised by the reviewer, we performed additional experiments to assess the behavior of M. abscessus subsp. massiliense and M. abscessus subsp. bolletii in airway organoids. All Mabs subspecies had similar growing rate in vitro in 7H9 medium (Fig EV1C). Interestingly, we showed that, M. abscessus subsp. bolletii displayed a higher growth rate in airway organoids compared to Mabs S and M. abscessus subsp. massiliense, which grew to the same extent (Fig EV1B). Further experiments will be required in the future to characterize the underlying mechanisms of M. abscessus subsp. bolletii fitness in the human airway context.*

*The reviewer also addressed a very relevant point regarding the drug sensitivity or resistance of M. abscessus in the organoid system. We agree that the organoid model requires now its validation for a potentially more predictive evaluation of the response of M. abscessus to treatment. The experiments require an appropriate selection of clinical isolates, fully characterized with patient follow-up and for their response to treatment in vitro, to be then further evaluated in the organoid system. In the context of this proof-of-concept study, we believe that such experiments are currently out of the scope of this revised manuscript. This important question is now under investigation in the lab, in collaboration with experts in the field and will give rise to future publication.*

Reviewer #2: In this study the authors establish airway organoids as a model to investigate the interactions between M. abscessus and the host epithelium. They generate organoids from “healthy” tissue as well as from cystic fibrosis patients and compare several parameters during infection with two distinct isolates of M. abscessus (a rough and a smooth one). This study is of interest for different reasons, including the description of the airway organoid model and its application during Mycobacterial infection, and the importance of non-tuberculous mycobacteria in the context of cystic fibrosis. Using this system the authors propose antioxidants as a potential host-directed strategy to improve M. abscessus infection control. Overall, the study is well designed and presented. Some aspects could be improved as pointed out below, mainly to advance the study beyond its descriptive nature.

*The authors thank the reviewer for his/her fair and constructive evaluation of our work.*

Reviewer #3: The manuscript by Leon-Icaza et al., exploited previously reported human airway organoids (H-OEs) to evaluate Mabs fitness, host redox responses to infection, and efficacy of antioxidants. They further examined OEs from cystic fibrosis (CF) patients and showed the importance of antioxidants and CFTR inhibitors in controlling ROS and bacterial fitness. Finally, the authors proposed a combination of antioxidant(s) with cefoxitin as a potential host-directed strategy to improve Mabs infection control. Overall, the manuscript is interesting from a technology point of view and infection control. However, several conclusions need supportive evidence. More importantly, mechanistic insights on how host redox mechanisms modulate Mab fitness are crucial.

*The authors thank the reviewer for his/her fair and constructive evaluation of our work. We now provide additional data to support our conclusion and new molecular insights. Please refer to our comments in Part II of this rebuttal letter.*

**Part II – Major Issues: Key Experiments Required for Acceptance**

Please use this section to detail the key new experiments or modifications of existing experiments that should be absolutely required to validate study conclusions.

Generally, there should be no more than 3 such required experiments or major modifications for a "Major Revision" recommendation. If more than 3 experiments are necessary to validate the study conclusions, then you are encouraged to recommend "Reject".

Reviewer #1: -

Reviewer #2: In Figure 1 the authors infect (microinject) airway organoids with two isolates of M. abscessus and demonstrate that the bacteria grows within the organoids over time. They chose to perform the experiments on day 4 post-infection. Looking at the CFU dynamics (Figure 1A), there is app a 1 log difference in bacterial burden between the two M. abscessus isolates used on day 4. Would this difference in bacterial burden impact the remaining analysis?

*Due to the difference in the initial bacterial load between Mabs S and R, the results initially presented in Figure 1A are now moved to Fig EV1A. The new Figure 1A presents data of CFU/organoids from 3 independent experiments showing bacterial load at day 0 and day 4. We found no statistical difference in bacterial load between S and R morphotypes right after microinjection (day 0). After 4 days of infection, we showed a significant lower bacterial load for the R morphotype (5282±* *1258 CFU/organoid) compared to the S one (13212±2413 CFU/organoid). As our study then showed that Mabs R induces higher oxidative stress and cell death in organoids, these phenotypes cannot be explained by a difference in bacterial burden between S and R morphotypes, but potentially by Mabs R capacity to form virulence structures such as serpentine cords and induces oxidative stress* [1–3]. *These data are now included in Figure 1D-E (page 4-5 of the revised manuscript).*

Also related to this first set of data, the authors describe several features of the bacterial distribution and interaction with the organoid cells, showing representative images. Quantifying these features would strengthen the data.

*Due to the 3D structure of the airway organoids and our current technical limitation to image a high number of whole organoids for the precise quantification of bacterial localization, it was not possible during the time of this revision process to robustly quantify the interactions of Mabs S with the epithelium. Approaches by light sheet imaging or electron microscopy of such 3D models mainly allowed us to provide descriptive information on the localization and aggregation of the bacteria. Nevertheless, we were able to provide additional illustrations of Mabs S phenotype in AOs (FigEV2A), and to quantify the cording phenotype of Mabs R using fluorescent bacteria and imaging under the EVOS M7000 microscope. We showed that only the R variant formed cords from day 2 post-infection, with an increasing number of organoids containing cords until day 4. The new data are now included in Figure 1E. We also dampened the wording related to Figure 1 (see page 5 of the revised manuscript).*

The authors then show that M. abscessus promotes a oxidative environment in the airway organoids. The rational for investigating specifically the oxidative environment, without considering other cellular responses was not clear. Is the oxidative response the most predominant one upon infection?

*Historically, we initiated our study by investigating AO responses to Mabs infection with the assessment of different host pathways. We evaluated by RT-qPCR genes related to inflammatory cytokines, anti-microbial peptides (AMPs), mucins, and redox pathways. These data are now included in Fig EV3A-C. We observed a slight modulation of AMP expression, and tendency towards the downregulation of genes related to cytokines and mucins, as previously described* [4]*. Among all the evaluated genes, those related to redox pathway, specifically the anti-oxidant master regulator NRF2 and its target gene NQO1 were highly expressed during Mabs R infection (Fig 2A). As redox pathways are altered in CF patients, we therefore focused the study on the role of ROS and antioxidant pathways during Mabs infection in CF organoid context. Data showing the expression of redox genes upon Mabs infection are now presented in the Figure 2A, and the focus on the redox pathways justified in the revised manuscript (see page 7).*

Furthermore, some differences are shown with respect to the two M. abscessus isolates, but the mechanisms possibly underlying these differences are not discussed- they are justified based on the differential virulence of the M. abscessus isolate.

Would the observed differences be for example linked to the differential structure of the smooth vs rough bacteria in the organoid, or their localization relatively to the epithelial cells? Providing some insights on these questions would advance the study beyond its descriptive nature.

*Difference between Mabs S and R morphotypes resides in the absence or downregulation of GPL at the cell surface of Mabs R, associated with higher exposure of trehalose-6,6-dimycolate (TDM) at the cell surface mediating the formation of serpentine cords. For other mycobacteria, such as M. tuberculosis and marinum that display only a rough morphotype, cord formation has been shown to correlate with virulence* *in patients* [2] *and in different animal models* [3,5]*. Here, using the airway organoid model, we confirm that Mabs R forms the extracellular serpentine cords (Fig 1B-E), induces higher level of oxidative stress and epithelial cell death than Mabs S (Fig 1F-G, Fig 2B-D). The cording phenotype of Mabs R was quantified in different experimental settings and our data show that the cording phenotype is increased over time of infection (Fig 1E), decreased upon antioxidant treatment (Fig 2G), and increased in CF context (Fig 4D). At the molecular level, we showed that Mabs R induces significantly higher level of the protective antioxidant response, revealed by the transcription factor* *nuclear factor erythroid-2-related factor 2 (NRF2) and the enhanced expression of the NRF2-regulated gene NQO1 (Fig 2A). All these new data are included in the revised manuscript (page 6).*

The airway organoids derived from cystic fibrosis patients recapitulated important features of the disease, namely the epithelial dysfunctions. The authors follow their study by comparing the M. abscessus infection in healthy vs cystic fibrosis organoids. It is not clear whether the microinjection efficiency is similar in both organoid types, as the CFU shown for day 0 relates only to the healthy data. Given the somehow different basal structure/characteristics of the healthy vs cystic fibrosis organoids, it would be interesting to address this point.

*In order to address the comment raised by the reviewer, we performed CFU assay on healthy and CF organoids harvested 2h after microinjection with Mabs to evaluate the initial bacterial load in each type of organoids. We measured no significant difference between the initial bacterial load in healthy versus CF organoids. We observed a tendency towards a reduced load in CF compared to healthy ones. These results indicate that the higher bacterial load of Mabs and cell death in CF organoids after 4 days of infection were not due to a higher number of bacteria initially injected in CF organoids, but rather reveal the higher growth of Mabs in CF context. These data are presented in Figure EV5A, and page 15 of the revised manuscript.*

Related to this, the propidium iodide incorporation in mock conditions is already different between the two types of organoids (Figure 4D,E), which makes it difficult to assess whereas the differences seen upon infection reflects “higher susceptibility of cystic fibrosis organoids to Mabs infection”. It may be worth to quantify the fold increase in cell death relatively to mock conditions.

*We performed an additional experiment to strengthen our results and, as suggested by the reviewer, normalized the quantification of propidium iodide incorporation in Mabs-infected healthy and CF organoids relative to their respective non-infected condition. The data are now included in Figure 4F, the original set of raw data is shown in FigEV5I. We confirm that Mabs infection by each morphotype induces significant cell death in both healthy and CF organoids. By normalizing to the non-infected condition for each type of organoids, we reveal that Mabs R induces higher level of cell death in CF organoids compared to healthy ones. As requested by reviewer 3, we also measured oxidative stress in healthy and CF AOs infected by each Mabs morphotype. In Figure 4E and Figure EV5J, we show that both Mabs S and R induced oxidative stress, which was significantly higher in CF context. Altogether, these data show that while both morphotypes induce higher oxidative stress in CF AOs, only Mabs R triggered higher cell death in CF context.*

Reviewer #3: It needs to be clarified how an increase in oxidative stress promotes bacterial fitness. In general, higher ROS is associated with better control of mycobacterial infection. R-form is also better equipped to induce ROS than S-form. Both of these observations need in-depth characterization. Is Mabs more tolerant to oxidative stress than Mtb? Comparative survival of Mab vs Mtb in response to oxidants in vitro is important.

*Due to the intracellular lifestyle of M. tuberculosis in macrophages, and the extracellular one of M. abscessus in the airway, we found the reviewer’s comment very relevant, but we believe that comparing the response to oxidative stress of Mtb versus Mabs is out of the scope of this study. In a previous work* [4]*, we compared Mtb with Mabs in airway organoid context. We showed that the AO environment was able to control, but not eliminate Mtb, while Mabs proliferated. In term of host responses, we showed that both mycobacteria downregulated expression of pro-inflammatory and mucin genes, while differentially modulating antimicrobial peptide expression, data we confirmed here with Mabs R. The objective of the current study is to decipher the pathogenicity of M. abscessus in the context of CF. As Mtb infection is not directly related to CF disease, we decided not introducing data with Mtb. Nevertheless, to address the reviewer comment, as we showed that the level of oxidative stress is a key parameter for the extracellular fitness of Mabs S and R, we compared the tolerance of Mabs S and R variants to oxidative stress. We measured Mabs survival in vitro under oxidative stress conditions induced by either the thiol-oxidizing agent diamide,* or *tert-Butyl hydroperoxide (tBHP) or Cumene hydroperoxide (CHP). Our results show that Mabs R is more resistant to diamide (5mM) than Mabs S, which is associated with higher intracellular redox stress in Mabs R than S, indicating that Mabs R variant is more resistant to oxidative stress than the S one. These data are now included in Fig EV6C and in the revised manuscript (page 8).*

The data need to be further supported by measuring the cytoplasmic redox potential of Mabs vs Mtb (or any other slow-growing mycobacteria that are known to infect macrophages). Similarly, experiments to show the cytoplasmic redox state of S-type vs R-type Mabs *in vitro* and *inside H-OEs from healthy and CF patients* are crucial to confirm improved bacterial fitness in response to higher oxidative stress.

*To address the comment raised by the reviewer, we developed Mabs strains expressing the redox sensitive fluorescent biosensor Mrx1-roGFP2* [6] *to measure mycothiol redox potential in response to oxidative stress in vitro and during organoid infection. For a matter of time, we focused these revision experiments on the comparison of Mabs S and R. Both variants were transformed to express Mrx1-roGFP2, incubated with Diamide, tBHP or CHP for 2h and the ratio between oxidized and reduced roGFP2 was quantified using a spectrofluorometer. First, at basal level, our results showed that the roGFP2ox/roGFP2red ratio is higher in Mabs S compared to Mabs R, indicative of a higher oxidized status in the S compared to the R variant. In order to compare variant responses to oxidative agents, the roGFP2ox/roGFP2red ratio of each morphotype in the non-treated conditions was normalized to 1. Upon exposure to oxidazing agents, we measured a slight increase in the roGFP2ox/roGFP2red of Mabs S, which was significantly higher for Mabs R, indicating that Mabs R displayed higher redox stress. Then, Mabs variants expressing Mrx1-roGFP2 were microinjected in healthy and CF organoids to assess bacterial redox stress in airway organoids. As a positive control of ROS induction, Mabs-infected organoids were treated with tBHP. We showed an increase in bacterial ROS in CF AOs compared to healthy ones, and higher ROS in R versus S in CF context. These results are now included in Fig 4H-I and Fig EV6A-B, D, and in the revised manuscript page 9.*

Synergy experiments between antioxidants and cefoxitin can be strengthened by examining if potentiation is only in the context of OGs. Measuring host ROS and bacterial redox state in response to antioxidants and cefoxitin combination compared to antioxidant/ cefoxitin alone further bolsters the findings.

*In order to measure bacterial ROS in vitro, we exposed the Mrx1-roGFP2 reporter strains of Mabs S and R to cefoxitin or sulforaphane or the 2 compounds together, in parallel diamide was used as a positive control of bacterial ROS induction. As shown in Fig EV7E, neither cefoxitin nor sulforaphane modulated bacterial redox status. Then, we assessed host redox level in CF organoids treated with cefoxitin or sulphorafane or the 2 compounds together upon Mabs S infection. As previously shown in healthy organoids (Fig 2B-D), Mabs S infection triggered ROS production in CF organoids. Interestingly, sulphorafane significantly reduced oxidative stress in Mabs-infected CF-AOs, to a higher extent than cefoxitine alone. Their combination did not reduce ROS level better than sulphorafane alone, indicating that the level of oxidative stress is mainly under the control of the NRF2 pathway. These results are now included in Fig 5E.*

Lastly, why does the R-type induces higher oxidative stress than the S-type?

*We thank the reviewer for this relevant comment that pushes our study beyond its initial results. The new set of data included in the revised manuscript strengthen that, compared to Mabs S, Mabs R induces more ROS and cell death correlating with enhanced bacterial cording. We identified that the expression of the oxidase DUOX1 was significantly higher upon Mabs R than Mabs S infection (Fig 2A). We also showed that the NRF2-regulated gene NQO1 displayed higher induction upon Mabs R infection compared to Mabs S (Fig 2A). Moreover, NQO1 expression is impaired in CF AOs, and sulforaphane treatment restored NQO1 expression, associated with reduced oxidative stress, and a better control of Mabs growth and reduced cording. These data suggest that the capacity of Mabs R to form cords might be associated to its capacity to induce ROS. Confirming the role of NQO1 in the control of Mabs growth, we showed that inhibition of NQO1 using dicoumarol abrogated sulforaphane effect and restored Mabs growth and cording in CF AOs (Fig 5C-D). Our study highlights the role of the host redox homeostasis and its imbalance in CF context to contribute to Mabs pathogenicity, especially the virulence of the R variant. Furthermore, Mabs R-induced mitoROS have been shown to trigger type 1 IFN signaling and cell death in macrophages* [7]*. Our results also show that Mabs R, inducing higher mtROS and cell death, triggered higher expression of the Interferon-stimulated gene CXCL10 (Fig EV2A), suggesting that a similar pathway might operate in epithelial cells, via an as-yet unknown mechanism. Indeed, how the R variant, lacking GPL expression and forming cords, triggers higher expression of DUOX1 and NQO1 and their respective role in epithelial cell redox balance and cell death will warrant further investigation.*

**Part III – Minor Issues: Editorial and Data Presentation Modifications**
Please use this section for editorial suggestions as well as relatively minor modifications of existing data that would enhance clarity.

Reviewer #1: Criticism: The entire investigation is conducted with one (set) of strains, namely M. abscessus type strain. It would be recommendable to extend the investigation to members of the other subspecies and to strains, which show increased/decreased susceptibility to the drug(s) used for treatment.

*As suggested by the reviewer, we compared the bacterial growth of Mabs to the other subspecies, here Mycobacterium abscessus massiliense and bollettii. Bacteria were microinjected in airway organoids, and bacterial load was measured 4 days post-infection. We showed that both Mycobacterium abscessus subspecie massiliense and subspecie bollettii grew in organoids, with bollettii displaying a higher growth than the two other species. These data are now included in Fig EV1B. The reviewer also addressed a very relevant point regarding the drug sensitivity or resistance of M. abscessus in the organoid system. We agree that the organoid model requires now further validation for its predictive evaluation of the response of M. abscessus to treatment. The requested experiments require an appropriate selection of clinical isolates, fully characterized with patient follow-up and for their response to treatment in vitro, to be then evaluated in the organoid system. In the context of this proof-of-concept study and this revision, we have felt that such experiments are out of the scope of this revised manuscript. This important question is currently under investigation in the lab, in collaboration with experts in the field and will give rise to future publication.*

Reviewer #2: Given the novelty of the model, it would be important for a general audience to provide more details in the methods section. For example, the section: “RT-qPCR and Colony forming unit assay. Organoids were collected at day 4 post-infection or stimulation and processed as reported (31).” does not provide any relevant information as to what and how has been done.

*As suggested by the reviewer, we extended the method section for RT-qPCR and CFU assays. Details are now included in the main manuscript (page 16) and in the Supplemental Information document.*

Despite the promising data shown in this study, and the potential of using airway organoids, the impact of immune cells in the system deserves a deeper discussion. It is true that the authors show a series of interesting findings even in the absence of immune cells, but their impact to the data needs further addressing.

*The absence of immune cells in the organoid system is now discussed of the revised manuscript (see page 12-13).*

Reviewer #3: Other comments.

Fig 1 A: Why are there differences at day 0 CFUs between S and R-types? Are there differences in internalization or initial establishment of infection?

*We agree that the set of data presented in the original Fig 1A showed an important variation in the initial bacterial load between Mabs S and R. This figure is now moved to Supp FigEV1A. The new figure 1A combines a new set of data and shows that organoids were microinjected with a comparable number of bacteria. After 4 days of infection, we showed that Mabs S grew better than Mabs R.*

Using the term latency to depict the lag shown by bacteria for the initial 2 days is inappropriate.

*The term “latency” has been removed and the sentence rephrased (see revised manuscript page 4)*

Fig. 1B/C/D” The images show clear bacteria. I am not convinced about the aggregate/biofilm nature of the S type or the serpentine nature of cords. Generally, cording improves with time and as bacteria lyse host cells and release in the outside milieu (extracellular). Necessary to show time-dependent changes in aggregate or cording behaviors? Quantification of cording needed (Cording/organoid)?

*As suggested by the reviewer, we strengthened our data on Mabs phenotype in AOs. We now provide additional electron microscopy illustrations of the extracellular polymeric substance surrounding Mabs S (see FigEV2A) and serpentine cords by Mabs R (FigEV2B). We also quantified bacteria cording in Mabs R-infected organoids overtime and showed an increase of the % of organoids containing bacteria cords between day 0 and day 4. Data are included in Figure 1E. We also show that the % of organoids containing Mabs R cords is increased in the CF context, and reduced after sulforaphane treatment. These data are now included in revised Figure 2G, 4D, EV5H, 5D.*

Fig. 1E/F shows the higher killing of epithelial cells, which cannot be equated to hypervirulence- better to avoid using hypervirulence to describe this phenomenon.

*According to the reviewer’s suggestion, the term “hypervirulence” was removed, and the sentence rephrased page 5 of the revised manuscript.*

Fig. 2A: Induction of ROS-producing and antioxidant genes higher in R-type infected OEs than S-type? Please clarify this observation in the results section.

*To address the review comment and clarify our results on ROS-producing and antioxidant gene expression in airway organoids upon infection with Mabs variants, we modified the presentation of the data. Now included in Fig 2A, we show that Mabs R induced higher expression of the ROS producing enzyme DUOX1 than Mabs S. Our results also show that while both Mabs S and R induced significant increase in NRF2 expression, only Mabs R induced a significant expression of the NRF2-regulated gene NQO1. As reflected by the measurement of ROS production in Mabs-infected organoids, we conclude that Mabs R induces a higher oxidative stress than Mabs S. These data are now included in the revised manuscript (page 6).*

Fig. 2B/D: Why were these experiments done after three days of infection, whereas other experiments at four days? tPBH is not a good choice for assessing mitoROS; better to use antimycin D or rotenone?

*As ROS production is transient, we performed mitoSOX staining earlier than CFU evaluation. To address the reviewer comment, we used antimycin A and rotenone as positive controls for mitoROS induction in healthy and CF AOs. Data are now included in Figure 3G.*

Fig 2E/H: What is the effect of antioxidants on the cording? This would be important to show if the reduced bacterial growth is due to direct interference with phase switching.

*As suggested by the reviewer, we quantified Mabs R cording after antioxidant treatment. We showed that in both healthy and CF airway organoids, the antioxidant sulforaphane reduced Mabs R cording. Data are included in Figure 2G and 5D.*

Why CF AOs images look different in Fig 3C vs 3F? In 3C, the size of CF-AOs looks bulkier, which is not the case with 3C.

*Size and epithelium thickness of healthy vs CF organoids are heterogeneous due to the culture condition in 3D matrix domes. Image in Fig 3F has been changed for another picture of a CF AO from the same experiment.*

Fig 3I-J needs a positive control.

*Cumene hydroperoxide treatment of healthy and CF AOs was included as a positive control of lipid peroxidation. The data are now included in the revised Figure 3J, EV4F.*

What is the effect of CFTR inhibitors on ROS and cytotoxicity upon infection?

*To address the reviewer comment, we evaluated microscopically the effect of CFTR inhibitors on ROS production and cytotoxicity using mitoSOX and propidium iodide respectively. We show that the treatment with CFTRinh was associated with an increase in ROS production and cell death, thus resulting in phenocopy of the CF patient-derived organoids displaying natural CFTR mutations. Interestingly, as observed using CF organoids, enhanced Mabs growth is again associated with higher oxidative stress and cell death, and enhanced cording of Mabs R, suggesting that inhibition of the CFTR is sufficient to favor Mabs growth and virulence. These data are now included in the FigEV5B-C.*

Fig. 4H: Survival of Mabs is reduced upon antioxidant and antibiotic combination (not inhibition?).

*The authors admit that they are not sure to fully understand the reviewer comment, but this part of the result section has been rephrase (pages 9 of the revised manuscript).*

Discussion: Cording also depends on other host factors (fh111, LTH4, TNf-Alpha)? These studies should be included in the discussion. Overall, the discussion could be more descriptive and more informative.

*According to the reviewer comment, studies describing the implication of host factors in mycobacteria cording has been included in the discussion of the revised manuscript (page 11).*

1.	Julián E, Roldán M, Sánchez-Chardi A, Astola O, Agustí G, Luquin M. Microscopic cords, a virulence-related characteristic of Mycobacterium tuberculosis, are also present in nonpathogenic mycobacteria. J Bacteriol. 2010;192: 1751–1760. doi:10.1128/JB.01485-09

2.	Ufimtseva EG, Eremeeva NI, Petrunina EM, Umpeleva T V., Bayborodin SI, Vakhrusheva D V., et al. Mycobacterium tuberculosis cording in alveolar macrophages of patients with pulmonary tuberculosis is likely associated with increased mycobacterial virulence. Tuberculosis. 2018;112: 1–10. doi:10.1016/J.TUBE.2018.07.001

3.	Tobin DM, Roca FJ, Oh SF, McFarland R, Vickery TW, Ray JP, et al. Host genotype-specific therapies can optimize the inflammatory response to mycobacterial infections. Cell. 2012;148: 434. doi:10.1016/J.CELL.2011.12.023

4.	Iakobachvili N, Leon-Icaza SA, Knoops K, Sachs N, Mazères S, Simeone R, et al. Mycobacteria-host interactions in human bronchiolar airway organoids. Mol Microbiol. 2021;00: 1–11. doi:10.1111/mmi.14824

5.	Bernut A, Herrmann JL, Kissa K, Dubremetz JF, Gaillard JL, Lutfalla G, et al. Mycobacterium abscessus cording prevents phagocytosis and promotes abscess formation. Proc Natl Acad Sci U S A. 2014;111: E943–E952. doi:10.1073/pnas.1321390111

6.	Bhaskar A, Chawla M, Mehta M, Parikh P, Chandra P, Bhave D, et al. Reengineering redox sensitive GFP to measure mycothiol redox potential of Mycobacterium tuberculosis during infection. PLoS Pathog. 2014;10. doi:10.1371/JOURNAL.PPAT.1003902

7.	Kim BR, Kim BJ, Kook YH, Kim BJ. Mycobacterium abscessus infection leads to enhanced production of type 1 interferon and NLRP3 inflammasome activation in murine macrophages via mitochondrial oxidative stress. PLoS Pathog. 2020;16. doi:10.1371/JOURNAL.PPAT.1008294

PLOS authors have the option to publish the peer review history of their article ([what does this mean?](https://journals.plos.org/plospathogens/s/editorial-and-peer-review-process#loc-peer-review-history)). If published, this will include your full peer review and any attached files.


If you choose “no”, your identity will remain anonymous but your review may still be made public.


**Do you want your identity to be public for this peer review?** For information about this choice, including consent withdrawal, please see our [Privacy Policy](https://www.plos.org/privacy-policy).

Reviewer #1: No

Reviewer #2: No

Reviewer #3: No
